# Association of baseline steroid use with long-term rates of infection and sepsis in the REGARDS cohort

**DOI:** 10.1186/s13054-017-1767-1

**Published:** 2017-07-13

**Authors:** Ninad S. Chaudhary, John P. Donnelly, Justin X. Moore, John W. Baddley, Monika M. Safford, Henry E. Wang

**Affiliations:** 1 Department of Emergency Medicine, University of Alabama School of Medicine, 619 19th Street South, OHB 251, Birmingham, AL 35249 USA; 2 Division of Preventive Medicine, Department of Medicine, University of Alabama School of Medicine, Birmingham, AL USA; 3 Department of Epidemiology, University of Alabama at Birmingham, Birmingham, AL USA; 4 Division of Infectious Disease, Department of Medicine, University of Alabama School of Medicine, Birmingham, AL USA; 5 Department of Medicine, University of Alabama at Birmingham, Birmingham, AL USA; 6 Department of Medicine, Weill Cornell Medical College, New York, NY USA; 7 Department of Emergency Medicine, University of Texas Health Science Center at Houston, 6431 Fannin St., JJL 434, Houston, TX 77030 USA

**Keywords:** Steroids, Infection, Longitudinal Study, Epidemiology, Prevention

## Abstract

**Background:**

Prior studies associate steroid use with infection risk but were limited to select populations and short follow-up periods. The association of steroid use with long-term risk of community-acquired infections is unknown. We sought to determine the association of steroid risk with long-term risks of community- acquired infections and sepsis.

**Methods:**

We used data on 30,239 adults aged ≥ 45 years old from the Reasons for Geographic and Racial Differences in Stroke (REGARDS) cohort. The primary exposure was oral or injectable steroid use, determined from medication inventory obtained at baseline in-home visit. The primary outcome was time to first infection event during 2003–2012, determined through adjudicated review of hospital records. We determined associations between baseline steroid use and first infection hospitalization events using Cox proportional hazards models, adjusting for demographics, health behaviors, chronic medical conditions, and medication adherence. Among the first infection hospitalization events, we also determined the association between baseline steroid use and sepsis.

**Results:**

Steroid use was reported in 2.24% (n = 677) of the study population. There were 2593 incident infection events during the 10-year follow-up period. Infection incidence rates were higher for steroid than non-steroid users (37.99 vs. 13.79 per 1000 person-years). Steroid use was independently associated with increased risk of infection (adjusted HR 2.10, 95% CI: 1.73–2.56). Among first-infection events, steroid use was associated with increased odds of sepsis (adjusted OR 2.11, 95% CI: 1.33–3.36). The associations persisted in propensity matched analyses as well as models stratified by propensity score and medication adherence.

**Conclusions:**

In this population-based cohort study, baseline steroid use was associated with increased long-term risks of community-acquired infections and sepsis.

**Electronic supplementary material:**

The online version of this article (doi:10.1186/s13054-017-1767-1) contains supplementary material, which is available to authorized users.

## Background

Community-acquired infections are a major public health problem. For example, community-acquired pneumonia accounts for 600,000–1.1 million hospitalizations and over $10 billion healthcare costs annually [1–4]. In many cases a serious community-acquired infection may progress to sepsis, the syndrome of microbial infection complicated by systemic inflammation and organ dysfunction. Sepsis is associated with significant morbidity, mortality (200,000 annual deaths), and substantial burden on national health care resources with annual medical expenditure of US$16.7 billion [5–7]. Recent studies suggest that identified baseline risk factors may offer opportunities for early sepsis risk detection or reduction [8].

**Fig. 1:**
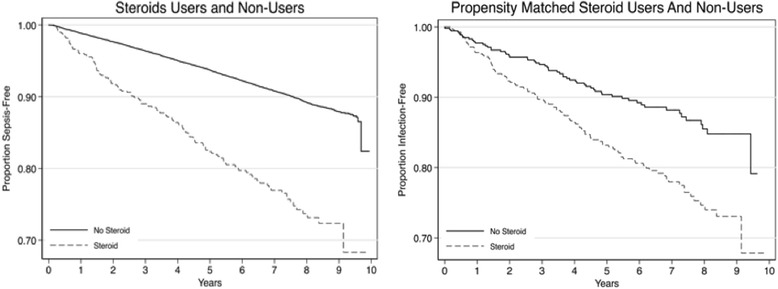
Kaplan-Meier curves depicting proportion of infection-free period among steroid users and non-users. *Left graph* depicts entire cohort population (n = 30,189). *Right graph* depicts steroid users and non-users matched on propensity score using nearest neighbor matching with caliper method (n = 1072)

Many individuals use systemic corticosteroids on a chronic basis; for example, for the treatment of asthma, autoimmune diseases such as rheumatoid arthritis, sarcoidosis, and vascular disorders. Chronic corticosteroids exposure is believed to increase the risk for infections due to long-term immunosuppression. Corticosteroid-induced immunosuppression may occur by inhibiting nitric oxide synthase, preventing adhesion of leukocytes induced by endotoxin, preventing activation of complement cascade, and preventing release of tumor necrosis factor, interleukin-1, and prostaglandins [9].

Prior studies have extensively linked steroids and infections [10]. However, these studies have focused on individuals with specific high-risk comorbidities in autoimmune diseases such as rheumatoid arthritis and targeted individual infections such as pneumonia [11–18]. Further, some of these studies determined steroid use retrospectively [19–21]. Few studies have characterized the risks of infection and sepsis from long-term steroid use among community-dwelling adults [21–24].

In this study we determined the association of baseline steroid use with long-term risks of infection and sepsis in the Reasons for Geographic and Racial Differences in Stroke (REGARDS) cohort, one of the nation’s largest population-based cohorts of community-dwelling adults [25]. We hypothesized that baseline use of steroids may be associated with increased long-term risk of serious infection and sepsis hospitalizations.

## Methods

### Study design

We used data from the Reasons for Geographic and Racial Differences in Stroke (REGARDS) study. The Institutional Review Board of the University of Alabama at Birmingham approved the study.

### The REGARDS cohort

REGARDS is one of the largest population-based prospective cohort studies in the United States. Designed to assess geographic and racial variations in stroke mortality, REGARDS is comprised of 30,239 community-dwelling individuals aged ≥ 45 years old. The cohort is 55% females, and 42% African American. Hispanics were not selected for this study. The study oversampled individuals from the Southeastern United States with 21% recruited from coastal plains of North Carolina, South Carolina, and Georgia, with 35% from the remaining areas of North Carolina, South Carolina, Georgia along with Tennessee, Mississippi, Alabama, Louisiana, and Arkansas.

Subject enrollment in REGARDS took place during 2003 to 2007. Subjects were randomly sampled, and recruited by mail for participation. Baseline information included medical history, functional status, health behaviors, height, weight, blood pressure, electrocardiogram, inventory of medications, dietary patterns, and family history of diseases, residential history, and psychosocial measures. The study collected urine and blood specimens from all individuals. The study contacted participants on a semi-annual basis to ascertain all hospitalizations and health care encounters. Death events during the follow-up period were reviewed separately to ascertain the cause and circumstances of death.

### Primary outcomes

The primary outcomes of the study were (1) hospitalization for a serious infection, and (2) among first-infection hospitalizations, the presence of sepsis. Two trained abstractors independently reviewed all relevant medical records to confirm the presence of serious infection and its relevance to the hospitalization. Reviewed medical records included emergency department physician and nursing notes, hospital admission notes, and initial laboratory test and vital signs, and the discharge summary. We defined serious infections based using the taxonomy described by Angus for identifying severe sepsis [5]. We did not use laboratory, microbiological, or radiographic information in defining serious infection.

We defined sepsis as hospitalization for infection plus ≥ 2 systemic inflammatory response syndrome criteria: (a) heart rate > 90 beats/min; (b) hyperthermia (>38.3 °C or < 36 °C); (c) respiratory rate (> 20 breaths/min) or PCO_2_ < 32 mm Hg; (d) white blood cell count > 12,000 or < 4000 cells/mm^3^ or > 10% band forms [5]. The criteria were verified from vital signs and laboratory reports for the initial 28 hours of hospitalization to encompass emergency care and 1 day of inpatient treatment. We excluded clinical information occurring after 28 hours of hospitalization. Independents abstractors reviewed all hospital records, with physician adjudication where there was disagreement. The chart abstractors were blinded to participant baseline characteristics, including baseline steroid use. Overall interrater agreement between two abstractors was 0.92 for hospitalization due to serious infection, and 0.90 for the presence of sepsis. We included all the first infection events that occurred between February 5, 2003 and December 31, 2012.

Recent consensus guidelines have proposed revised definitions for sepsis based upon the presence of organ dysfunction (Sepsis-3) [26]. To maintain consistency with our prior works, we opted to define sepsis using the older systemic inflammatory response syndrome (SIRS) definition.

### Primary exposure

The primary exposure was baseline systemic steroid use. Participants provided information on medication usage at the time of enrollment in REGARDS. As part of the subject enrollment process, study personnel documented all medications taken by study participants, reviewing pharmacy labels for all medications used by the participant in the prior 2 weeks. We defined baseline steroid use as the reported use of oral or injectable hydrocortisone, dexamethasone, fludrocortisone, prednisone, methyl prednisone, budesonide, and stanozolol. Because of their lesser systemic effects, we did not include topical or inhaled steroids [27]. We could not ascertain changes in medication use after the baseline interview.

### Other covariates

Sociodemographic variables included in the analysis included age, sex, race (blacks, whites), education (less than high school, high school, some college, and college or higher education), and annual income. Health behaviors included smoking status (current, past, never) and alcohol use over the past month (none, moderate, heavy).

Chronic medical conditions included self-reported history of stroke, history of cardiovascular events, renal comorbidities, vascular comorbidities, chronic lung disease, dyslipidemia, diabetes, and obesity. Cardiovascular comorbidities included myocardial infarction, hypertension, atrial fibrillation, and coronary artery disease. Myocardial infarction and atrial fibrillation were determined from medical history or positive findings on electrocardiogram (EKG). Hypertension was determined by self-reported use of anti-hypertensive drugs, or blood pressure measurements. Coronary artery disease was established by self-reported history of MI, coronary intervention, or baseline evidence of myocardial infarction on EKG. Chronic kidney disease was defined as measured glomerular filtration rate of < 60 ml/min based upon serum creatinine.

Vascular comorbidities included self-reported peripheral artery disease, and deep vein thrombosis. Chronic lung disease (CLD) was determined by reported use of pulmonary medications. Dyslipidemia was diagnosed based on participants who self-reported high cholesterol, or the use of anti-lipid agents. Diabetes was based on the use of anti-diabetic medications or blood glucose measurement > 125 mg/dl. Obesity was assessed using waist circumference (WC) or body mass index (BMI). Both WC and BMI were measured at the beginning of the study. WC was measured in standing position midway between the lowest rib and the iliac crest, with normal WC defined as ≤ 102 cm for males and ≤ 88 cm for females and large WC as > 102 cm for males, and > 88 cm for females. We categorized BMI as underweight (< 18.5 kg/m^2^), normal (18.5–25.0 kg/m^2^), overweight (25.0–29.9 kg/m^2^), obese (30–39.9 kg/m^2^), and morbidly obese (≥ 40 kg/m^2^) [28].

We assessed compliance with medication use using self-reported Morisky Medication Adherence Scale administered at the baseline [29]. The Morisky score categories included good (score = 0), fair (score = 1), and poor (score = 2 to 4).

### Data analysis

We compared subject characteristics, and covariates between steroid users and steroid non-users using Pearson chi-square test for categorical variables and independent *t* tests for continuous variables. We fit Cox proportional hazard models to assess the association of steroid use with time to first events of infections. Participants were censored on loss to follow-up or death. We adjusted the models for socio-demographics, health behaviors, chronic medical condition, and Morisky medication adherence.

We verified proportionality assumptions using Schoenfeld residuals assessing the time × steroid interaction. Because the risk of steroid use appeared to vary with time, we also fit a piecewise Cox regression model based the follow-up time intervals of 0–1 year, 1–2 years, 2–5 years, and 5–10 years.

To verify the robustness of the findings, we repeated analysis stratified by Morisky medication adherence scores. We also repeated the analysis stratified by propensity for steroid use. We included age, race, income, education, alcohol use, smoking status, atrial fibrillation, chronic kidney disease, chronic lung disease, coronary artery disease, deep vein thrombosis, diabetes, dyslipidemia, hypertension, myocardial infarction, obesity, peripheral artery disease, stroke, Morisky Adherence Scale, and high sensitivity C-reactive protein in the definition of the propensity score. We defined a non-parsimonious propensity model of covariates which satisfied balancing property. In addition, we repeated the analysis using 1:1 nearest neighbor propensity score matching within fixed caliper width [30].

Among those experiencing an infection hospitalization, chronic steroid use may confer additional risks of sepsis. To test this association, we fit multivariable logistic regression models limited to participants experiencing a first infection event. We adjusted this model for demographics (age, race, income, education, income), health behaviors (alcohol use, smoking status), chronic medical conditions (atrial fibrillation, chronic kidney disease, chronic lung disease, coronary artery disease, deep vein thrombosis, diabetes, dyslipidemia, hypertension, myocardial infarction, obesity, peripheral artery disease, stroke), and Morisky Adherence Scale. All analyses were performed using Stata v.14.1 (Stata Corp., College Station, TX, USA).

## Results

Among 30,239 participants in the REGARDS, 677 (2.24%) reported baseline systemic steroid use. Compared to non-users, steroid users were more likely to be white, female and reported lower education and annual income. Steroid users were more likely to be non-smokers and non-user of alcohol, but had an overall higher number of comorbidities (Table 1).

**Table 1:** Characteristics of REGARDS participants, stratified by steroid use

Characteristic	Steroid user (n = 677)	Steroid non-user (n = 29,506)	*P* value
Demographics			
Age (mean ± SD)	65.7 (9.4)	64.8(9.4)	0.01
Sex			< 0.001
Male	255 (37.7)	13,296 (45.1)	
Female	422 (62.3)	16,210 (54.9)	
Race			0.08
White	374 (55.2)	17,295 (58.6)	
Black	303 (44.8)	12,211 (41.4)	
Income			< 0.001
< $20,000	178 (26.3)	5300 (17.9)	
$20,000–34,000	191 (28.2)	7116 (24.1)	
$35,000–74,000	165 (24.4)	8749 (29.7)	
≥ $75,000	61(9.0)	4693 (15.9)	
Unknown	82 (12.1)	3648 (12.4)	
Education			0.002
Less than high school	103 (15.2)	3689 (12.5)	
High school graduate	193 (28.5)	7611 (25.8)	
Some college	193 (28.5)	7897 (26.8)	
College or higher	188 (27.8)	10,284 (34.8)	
Region			0.78
Belt	235 (34.7)	10,212 (34.6)	
Buckle	148 (21.8)	6159 (20.9)	
Non-belt	294 (43.4)	13,135(44.5)	
Health behaviors			
Tobacco use			0.233
Never	293 (43.3)	11,774 (39.9)	
Past	291 (42.9)	13,313 (45.1)	
Current	89 (13.2)	4307 (14.6)	
Missing (116)	4 (0.6)	112(0.4)	
Alcohol use			< 0.001
None	465 (68.7)	18,082 (61.3)	
Moderate	181 (26.7)	9675 (32.8)	
Heavy	14 (2.1)	1173 (3.9)	
Missing (593)	17 (2.5)	573 (2.0)	
Chronic medical conditions			
Atrial fibrillation	100 (14.8)	2493 (8.4)	< 0.001
Chronic kidney disease	131 (19.3)	3160 (10.7)	< 0.001
Chronic lung disease	169 (24.9)	2596 (8.8)	< 0.001
Coronary artery disease	159 (23.5)	5155 (17.5)	< 0.001
Deep vein thrombosis	55 (8.1)	1527 (5.2)	0.003
Diabetes	191 (28.2)	6623 (22.4)	0.001
Dyslipidemia	348 (51.4)	16,880 (57.2)	< 0.001
Hypertension	454 (67.1)	17,393 (58.9)	< 0.001
Myocardial infarction	112 (16.5)	3661 (12.4)	0.004
Obesity(abnormal BMI or waist circumference)	385 (56.9)	15,758 (53.4)	0.20
Peripheral artery disease	21 (3.1)	651 (2.2)	0.12
Stroke	56 (8.3)	1874 (6.4)	0.13
Morisky Medication Adherence Scale			< 0.001
0 (good)	463 (68.4)	18,786 (63.7)	
1 (fair)	130 (19.2)	5937 (20.1)	
2–4 (poor)	67 (9.9)	2020 (6.9)	

Among cohort participants, 2593 experienced hospitalizations for a serious infection. Median follow-up time was 6.6 years (IQR 5.1–8.1). The hazard of serious infection was twice as high for steroid users as non-users (adjusted HR 2.10; 95% CI 1.73–2.56, Fig. 1). These associations persisted with stratification by Morisky medication adherence and propensity for steroid use (Table 2). When we matched participants by propensity for steroid use, we observed similar associations (Table 3, Fig. 1). There was no potential effect modification observed which was confirmed by non-significant interactions of covariates with steroid use.

**Table 2:** Multivariable hazard ratios model evaluating association between steroid use and infections stratified by Morisky Adherence Scale and propensity scores

Model	Crude hazard ratio (95% CI)	Adjusted hazard ratio (95% CI)
Full cohort (n = 29,683)	2.78 (2.33– 3.31)	2.10 (1.73–2.56)
Stratified by propensity scores^a^		
Low propensity for steroid use	3.26 (1.95–5.46)	3.39 (2.02–5.69)
Medium propensity for steroid use	1.86 (1.19–2.90)	1.81 (1.15–2.82)
High propensity for steroid use	2.20 (1.72–2.81)	2.02 (1.58–2.60)
Stratified by Morisky Medication Adherence^b^		
Good Medication Adherence	2.97 (2.41–3.66)	2.16 (1.70–2.77)
Fair Medication Adherence	2.07 (1.36–3.13)	1.83 (1.17–2.89)
Poor Medication Adherence	2.38 (1.38–4.09)	2.79 (1.54–5.05)

^a^Models adjusted for demographics (age, race, income, education, income), health behaviors (alcohol use, smoking status, chronic medical conditions (atrial fibrillation, chronic kidney disease, chronic lung disease, coronary artery disease, deep vein thrombosis, diabetes, dyslipidemia, hypertension, myocardial infarction, obesity, peripheral artery disease, stroke), Morisky Adherence Scale

^b^Model adjusted as stated above except Morisky Adherence Scale was replaced by propensity scores

**Table 3:** Multivariable Cox regression model evaluating association between steroid use and first infection events

Exposure	Total N	Event N (%)	IR per 1000 py (95% CI)	Crude	Model 1	Model 2	Model 3
				HR (95% CI)	HR (95% CI)	HR (95% CI)	HR (95% CI)
Baseline chronic steroid use – full cohort							
Steroid user	677	132 (19.5)	37.99 (32.03–45.06)	2.78 (2.33– 3.31)	2.64 (2.22–3.15)	2.60 (2.17–3.10)	2.10 (1.73–2.56)
Non-user	29,506	2,461 (8.5)	13.79 (13.26–14.35)	Ref	Ref	Ref	Ref
Baseline chronic steroid use – propensity matched^a^							
Steroid user	538	103 (19.2)	36.37 (29.99–44.12)	1.89 (1.38–2.60)	1.97 (1.43–2.72)	1.98 (1.44–2.73)	2.01 (1.45–2.78)
Non-user	538	60 (11.2)	19.34 (15.01–24.91)	Ref	Ref	Ref	Ref

Model 1 = adjusted for demographics (age, race, income, education, income); Model 2 = Model 1 + health behaviors (alcohol use, smoking status); Model 3 = Model 2 + chronic medical conditions (atrial fibrillation, chronic kidney disease, chronic lung disease, coronary artery disease, deep vein thrombosis, diabetes, dyslipidemia, hypertension, myocardial infarction, obesity, peripheral artery disease, stroke), Morisky Adherence Scale

*IR* incidence rate, *PY* person years

^a^Propensity score includes age, race, income, education, income, alcohol use, smoking status, atrial fibrillation, chronic kidney disease, chronic lung disease, coronary artery disease, deep vein thrombosis, diabetes, dyslipidemia, hypertension, myocardial infarction, obesity, peripheral artery disease, stroke, Morisky Adherence Scale, and C-reactive protein

We repeated the analysis by fitting a piecewise model over sequential 2-year time segments. The risk of infection was five times higher among steroid users during the first year of follow-up, decreasing to 2.3 times at the end of 10 years of follow-up (Additional file 1).

Among the 2593 first serious infection hospitalizations, 1526 met sepsis criteria. In the serious infection subgroup, the odds of sepsis were twice as high for steroid users as non-users (Table 4). The associations were persistent when stratified by propensity score, and Morisky Medication Adherence Scale (Additional file 2).

**Table 4:** Multivariable logistic regression model evaluating association between steroid use and sepsis events nested within first infection events

	Sepsis	No sepsis	Odds ratios (95% CI)			
Baseline steroid use	N (%)	N (%)	Unadjusted	Adjusted		
				Model 1	Model 2	Model 3
Steroid user	89 (6.4)	35 (3.3)	2.03 (1.36–3.03)	2.09 (1.40–3.14)	2.04 (1.36–3.07)	2.11 (1.33–3.36)
Non-user	1299(93.6)	1039 (96.7)	Ref	Ref	Ref	Ref

Model 1 = Adjusted for demographics (age, race, income, education, income); Model 2 = Model 1 + health behaviors (alcohol use, smoking status); Model 3 = Model 2 + chronic medical conditions (atrial fibrillation, chronic kidney disease, chronic lung disease, coronary artery disease, deep vein thrombosis, diabetes, dyslipidemia, hypertension, myocardial infarction, obesity, peripheral artery disease, stroke), Morisky Adherence Scale

## Discussion

In this study we observed an independent association between baseline steroid use and long-term risk of community-acquired infections. We also found that among first infection events, baseline steroid use was also independently associated with the presence of sepsis. The magnitude of these associations was large and persisted with a range of sensitivity analyses. Our observations are based upon comprehensive data from the large population-based REGARDS cohort and add to the evidence linking steroid use with infection and sepsis risk.

Prior studies have characterized the association between steroid use and infection risk but in narrower subsets and over shorter observation periods. In a study of 95 persons, Rello et al. observed that immunosuppression was associated with increased risk of community-acquired pneumonia. However, the steroid use was not clearly defined, and the observed association was not statistically significant [15]. Other studies focused on high-risk populations such or those with rheumatoid arthritis or admitted to the intensive care unit [13, 16, 18]. Our study evaluated community-dwelling adults at a stable phase of health, included a range of community-acquired infections, and encompassed almost 10 years of follow-up.

In analyses of medication use, confounding by indication may influence the results, resulting in high medication use among those with multiple comorbidities. Our study affirmed this possibility, as there were a higher numbers of comorbid conditions among steroid users. Such biases have frequently influenced results of observational studies on medication usage or outcomes [31]. We tried to overcome this potential bias by using propensity score adjustment, stratification and matching, finding consistent results regardless of analytic strategy [32]. However, other unmeasured factors may drive the association between steroid use and infection risk. Additional studies must clarify these relationships.

Our study is unique because we could characterize the long-term risk of sepsis owing to steroid use. These findings are clinically important from two perspectives. First, these observations emphasize that steroid users are more susceptible to community-acquired infections aftermath to usage, and require frequent monitoring. Second, steroid use is a modifiable risk factor; discontinuation of steroid use may be considered when the risk for infections is heightened.

An important observation was that the risk of serious infection and sepsis were higher among those with low propensity for steroid use. We believe that these observations are due to the multiple comorbidities of higher propensity subjects, which was probably associated with higher baseline risk of infection and sepsis. Hence, the additional use of steroids may have a more profound effect upon infection and sepsis risk in those with low propensity for steroid use. Another important observation in our study was the stronger association with baseline steroid at earlier rather than later periods. Steroid use may not be persistent for the entire time period. The risk of sepsis in the early period may be due to the acute effects of steroid exposure rather than chronic exposure. Another possibility is that the adverse effect of chronic steroid use may stabilize over time. Additional confounders may have also developed over the course of the observation period. While we obtained baseline measures of comorbidities, we could not examine changes over time.

Our study has important limitations. Our observed associations cannot indicate a causal relationship between steroid use and the risk of serious infections or sepsis. While we adjusted for a range of covariates, there is a possibility of residual confounding from unmeasured variables. We had no information on the pattern or duration of steroid use over the follow-up period. We could not differentiate episodic from long-term chronic steroid use. We did not study more severe outcomes associated with infections and sepsis such as severe sepsis, sepsis-associated complications, and sepsis-related case fatality. We defined sepsis using the 2001 International Consensus definition, instead of the updated Sepsis 3 definition [26, 33]. Though we cannot determine here the direction of the association between steroid use and sepsis defined using newer guidelines, it has been reported that new sepsis-sequential organ failure assessment (SOFA) classification identifies patients with infection who are at increased risk of poor outcomes [34]. The assessment of community factors such as anti-microbial resistance, or health care access was beyond the scope of this analysis. We relied on self-reported history of hospitalizations, steroid use and medication adherence, which may be subject to recall or reporting bias. We did not include other immunosuppressive medications in the analysis.

## Conclusions

In this study of community-dwelling adults, baseline steroid use was associated with increased long-term risks of infections and sepsis.

## Additional files

Additional file 1: Table reporting piecewise Cox regression model evaluating association between steroid use and first infection events stratified by follow-up time stratified by time in study. (PDF 198 kb)

Additional file 2: Table reporting multivariable logistic regression model evaluating association between steroid use and sepsis events stratified by Morisky Adherence Scale and propensity scores. (PDF 181 kb)

## Abbreviations

BMI: body mass index
ECG: electrocardiogram
REGARDS: Reasons for Geographic and Racial Differences in Stroke
WC: waist circumference
