# Supplementary material for: Association of baseline steroid use with long-term rates of infection and sepsis in the REGARDS cohort
Source: Crit Care. 2017 Jul 13;21:185. doi: 10.1186/s13054-017-1767-1 (PMC5508766; doi:10.1186/s13054-017-1767-1)
Supplement: Supplementary file 1 — Table reporting piecewise Cox regression model evaluating association between steroid use and first infection events stratified by follow-up time stratified by time in study. (PDF 198 kb) [file 13054_2017_1767_MOESM1_ESM.pdf]

APPENDIX 1: Piecewise Cox Regression Model Evaluating Association between Steroid Use and First Infection Events stratified by follow up time stratified by time in study

Time Intervals	Exposure	Total N	Event N	IR per 1000 py (95% CI)	Crude HR (95% CI)	Model 1 HR (95% CI)	Model 2 HR (95% CI)	Model 3 HR (95% CI)
**0 -1 years**	Steroid Non-User	29021	199	6.93 (6.03-7.97)	**Ref**	**Ref**	**Ref**	**Ref**
	Steroid User	669	23	35.57 (23.64-53.52)	5.14 (3.34-7.93)	4.85 (3.14-7.48)	4.65 (2.99-7.24)	2.92 (1.73-4.90)
**1-2 years**	Steroid Non-User	28229	215	7.82 (6.84-8.93)	**Ref**	**Ref**	**Ref**	**Ref**
	Steroid User	618	13	22.00 (12.77-37.89)	2.82 (1.61-4.93)	2.60 (1.48-4.56)	2.47 (1.38-4.43)	1.93 (1.01-3.70)
**2-5 years**	Steroid Non-User	26994	688	9.30 (8.64-10.03)	**Ref**	**Ref**	**Ref**	**Ref**
	Steroid User	566	44	30.16 (22.44-40.53)	3.26 (2.40-4.42)	3.16 (2.32-4.29)	3.11 (2.27-4.25)	2.39 (1.67-3.41)
**> 5 years**	Steroid Non-User	22312	1365	26.49 (25.12-27.93)	**Ref**	**Ref**	**Ref**	**Ref**
	Steroid User	412	53	58.55 (44.73-76.64)	2.30 (1.75-3.03)	2.19 (1.67-2.89)	2.20 (1.67-2.91)	1.79 (1.32-2.44)

Model 1 = Adjusted for demographics (age, race, income, education, income); Model 2 = Model 1 + Health Behaviors (Alcohol use, Smoking Status); Model 3 = Model 2 + Chronic Medical conditions (atrial fibrillation, Chronic Kidney Disease, Chronic Lung Disease, Coronary Artery Disease, Deep Vein Thrombosis, Diabetes, Dyslipidemia, Hypertension, Myocardial Infarction, Obesity, Peripheral Artery Disease, Stroke), Morisky Adherence Scale

APPENDIX 2: Multivariable Logistic Regression Model Evaluating Association between Steroid Use and Sepsis Events Stratified by Morisky Adherence Scale and Propensity Scores

Model	Crude Odds Ratio (95% CI)	Adjusted Odds Ratio (95% CI)
**Full Cohort (n= 2,600)**	2.03 (1.36-3.03)	2.11 (1.33-3.36)
**Stratified by Propensity Scores [a]**		
Low Propensity for steroid use	4.82 (1.07-21.69)	5.68 (1.18-27.29)
Medium Propensity for steroid use	2.24 (0.92-5.43)	2.51 (1.00- 6.29)
High Propensity for steroid use	1.78 (0.99-3.17)	1.72 (0.93- 3.19)
**Stratified by Morisky Medication Adherence [b]**		
Good Medication Adherence	2.34 (1.44-3.79)	2.49 (1.38-4.50)
Fair Medication Adherence	1.67 (0.66-2.14)	1.71 (0.62-4.74)
Poor Medication Adherence	1.42 (0.42- 4.81)	2.40 (0.50-11.55)

[a] Models adjusted for demographics (age, race, income, education, income), Health Behaviors (Alcohol use, Smoking Status, Chronic Medical conditions (atrial fibrillation, Chronic Kidney Disease, Chronic Lung Disease, Coronary Artery Disease, Deep Vein Thrombosis, Diabetes, Dyslipidemia, Hypertension, Myocardial Infarction, Obesity, Peripheral Artery Disease, Stroke), Morisky Adherence Scale

[b] Model adjusted as stated above except Morisky Adherence Scale was replaced by Propensity Scores
