# Supplementary material for: Association of baseline steroid use with long-term rates of infection and sepsis in the REGARDS cohort
Source: Crit Care. 2017 Jul 13;21:185. doi: 10.1186/s13054-017-1767-1 (PMC5508766; doi:10.1186/s13054-017-1767-1)
Supplement: Supplementary file 2 — Table reporting multivariable logistic regression model evaluating association between steroid use and sepsis events stratified by Morisky Adherence Scale and propensity scores. (PDF 181 kb) [file 13054_2017_1767_MOESM2_ESM.pdf]

APPENDIX 2: Multivariable Logistic Regression Model Evaluating Association between Steroid Use and Sepsis Events Stratified by Morisky Adherence Scale and Propensity Scores

Model	Crude Odds Ratio (95% CI)	Adjusted Odds Ratio (95% CI)
**Full Cohort (n= 2,600)**	2.03 (1.36-3.03)	2.11 (1.33-3.36)
**Stratified by Propensity Scores [a]**		
Low Propensity for steroid use	4.82 (1.07-21.69)	5.68 (1.18-27.29)
Medium Propensity for steroid use	2.24 (0.92-5.43)	2.51 (1.00- 6.29)
High Propensity for steroid use	1.78 (0.99-3.17)	1.72 (0.93- 3.19)
**Stratified by Morisky Medication Adherence [b]**		
Good Medication Adherence	2.34 (1.44-3.79)	2.49 (1.38-4.50)
Fair Medication Adherence	1.67 (0.66-2.14)	1.71 (0.62-4.74)
Poor Medication Adherence	1.42 (0.42- 4.81)	2.40 (0.50-11.55)

[a] Models adjusted for demographics (age, race, income, education, income), Health Behaviors (Alcohol use, Smoking Status, Chronic Medical conditions (atrial fibrillation, Chronic Kidney Disease, Chronic Lung Disease, Coronary Artery Disease, Deep Vein Thrombosis,
Diabetes, Dyslipidemia, Hypertension, Myocardial Infarction, Obesity, Peripheral Artery Disease, Stroke), Morisky Adherence Scale

[b] Model adjusted as stated above except Morisky Adherence Scale was replaced by Propensity Scores
